# Real-world effectiveness and safety of finerenone in diabetic kidney disease with preserved eGFR: a retrospective study in China

**DOI:** 10.1186/s12882-026-05035-4

**Published:** 2026-05-12

**Authors:** Yiru Xu, Sha Zeng, Yanhua Xi, Cuiyun Chen, Guilin Zou, Yan Zhang, Jianying Liu

**Affiliations:** 1 Department of Endocrinology and Metabolism, The First Affiliated Hospital of Nanchang University, Nanchang, Jiangxi 330006 China; 2 Jiangxi Clinical Research Center for Endocrine and Metabolic Disease, Nanchang, Jiangxi 330006 China; 3 Jiangxi Branch of National Clinical Research Center for Metabolic Disease, Nanchang, Jiangxi 330006 China

**Keywords:** Finerenone, Diabetic kidney disease, Proteinuria, Safety, Urine albumin-to-creatinine ratio, Estimated glomerular filtration rate

## Abstract

**Objective:**

Given the limited real-world evidence on finerenone in patients with diabetic kidney disease (DKD), particularly with preserved renal function, this study aimed to evaluate the effectiveness and safety of finerenone in DKD patients.

**Methods:**

We retrospectively analyzed 127 patients with DKD treated with finerenone, including those with preserved renal function. Changes from baseline in the urine albumin-to-creatinine ratio (UACR), urinary microalbumin (mALB), urinary protein semi-quantitative, urinary N-acetyl-β-D-glucosaminidase (NAG) and estimated glomerular filtration rate (eGFR) were evaluated at 1, 3, and 6 months post-treatment. The safety profile was recorded during treatment.

**Results:**

Following treatment with finerenone, the median UACR was significantly reduced from a baseline of 100 mg/g to 59 mg/g, 48 mg/g, and 43 mg/g at 1, 3, and 6 months, respectively (*p* < 0.001 for 1 and 3 months). Similarly, mALB levels significantly decreased over the same period (*p* < 0.001). Stratified analysis revealed that the mean UACR reduction at 1, 3, and 6 months was 35%, 33%, and 21% in stage A2 patients, while it was 44%, 58%, and 57% in stage A3 patients (*p* < 0.0001). Moreover, finerenone significantly improved UACR staging classification, while also reducing both the proportion of proteinuria (*p* < 0.0001) and urinary NAG levels (*p* = 0.001). In terms of safety, eGFR exhibited an initial decline followed by a subsequent rise (*p* = 0.117). Three patients (2.4%) experienced hyperkalemia, and one patient discontinued treatment due to a decrease in eGFR of more than 30%.

**Conclusion:**

Finerenone demonstrated a significant reduction in proteinuria in DKD patients, particularly in patients with UACR stage A3, with a favorable safety profile.

**Supplementary Information:**

The online version contains supplementary material available at 10.1186/s12882-026-05035-4.

## Introduction

Diabetic kidney disease (DKD), a critical microvascular complication of diabetes, is characterized by persistent proteinuria and a progressive decline in the estimated glomerular filtration rate (eGFR) that can culminate in end-stage renal disease (ESRD) [1, 2]. The International Diabetes Federation reported that 1 in 9 adults (589 million) globally are living with diabetes, and this increasing pandemic has established DKD as the leading cause of chronic kidney disease (CKD) and ESRD worldwide, imposing a substantial clinical and economic burden on healthcare systems [3]. The presence of proteinuria, even with a normal or near-normal eGFR, is a key risk factor for the progression of renal damage [4]; therefore, early intervention is critical to preserve long-term kidney function.

The treatment of DKD has expanded beyond glycemic and blood pressure control to a multi-faceted approach targeting specific pathophysiological mechanisms, now including renin-angiotensin system inhibitors (RASi), sodium-glucose co-transporter type 2 inhibitors (SGLT-2i), and mineralocorticoid receptor antagonists (MRA) [5]. One of the drivers in the progression of DKD is the hyperactivation of the mineralocorticoid receptor (MR), a nuclear transcription factor that promotes pro-inflammatory and pro-fibrotic gene expression, leading to inflammation and fibrosis in the kidney and heart [6, 7]. While traditional steroidal MRAs like spironolactone reduce albuminuria, their clinical utility is hampered by a significant risk of hyperkalemia [8–10].

Finerenone is a novel, selective, nonsteroidal MRA with a unique chemical structure that confers high affinity for the MR with a distinct antagonistic profile compared to steroidal MRAs. This results in potent anti-inflammatory and anti-fibrotic effects with a lower propensity for causing hyperkalemia [11]. Pivotal randomized controlled studies (RCTs) like FIDELIO-DKD and FIGARO-DKD have unequivocally demonstrated the cardiorenal benefits of finerenone across a broad spectrum of patients with type 2 diabetes mellitus (T2DM) and CKD, leading to its inclusion in expert consensus guidelines as standard of care [12–15]. Although the evidence from trials has been robust, it is necessary to know how finerenone fares in routine clinical practice as trials possess rigorous inclusion/exclusion criteria, which create a highly selected group of patients who may fail to reflect the heterogeneity of patients in real-world practice [16]. These trials largely excluded patients with preserved renal function (eGFR ≥ 90 mL/min/1.73 m²), creating a need for evidence in this specific population. Recently, several retrospective real-world studies have been reported [17–19], providing information on the effectiveness and safety of finerenone in different healthcare systems and patient populations. However, these studies were not specifically designed to investigate finerenone’s role in the DKD patients, particularly in preserved renal function patients. There is still a lack of data to support the effectiveness and safety of finerenone among such patients.

Therefore, the aim of this study was to assess the effectiveness and safety of finerenone in the management of DKD patients and preserved renal function patients in a Chinese real-world scenario.

## Methods

### Study design and patients

This was a retrospective, observational study involving patients with DKD who received finerenone at the outpatient clinic of the Department of Endocrinology and Metabolism at the First Affiliated Hospital of Nanchang University between March 2023 and April 2024. This retrospective study was conducted in accordance with the Declaration of Helsinki. The requirement for informed consent was waived by the Ethics Committee due to the retrospective nature of the study. The study was approved by the Ethics Committee of the First Affiliated Hospital of Nanchang University (Approval No.: IIT [2024] Clinical Ethical Review No. 634).

Patients with a diagnosis of DKD based on the Clinical Guideline for the Prevention and Treatment of Diabetic Kidney Disease in China (2021 edition) [20] were included. According to the guideline, after identifying diabetes as the cause of renal impairment and excluding other factors contributing to CKD, at least one of the following conditions must be met: Urine albumin-to-creatinine ratio (UACR) ≥ 30 mg/g or urinary albumin excretion rates of ≥ 30 mg/24 h (≥ 20 µg/min) in at least 2 out of 3 tests conducted within 3 to 6 months, without any interfering factors; an eGFR of < 60 mL/(min 1.73m^2^) for more than 3 months; a renal biopsy showing pathological changes consistent with DKD. The exclusion criteria were as follows: UACR < 30 mg/g; eGFR ≤ 25 mL/ (min 1.73 m²); pregnancy or lactation.

### Data collection and definition

Clinical data were obtained from patients’ outpatient electronic medical records. Finerenone was added to the existing glycemic control, hypertension, and dyslipidemia, at the discretion of the treating physician. Baseline data were collected from patients at the time of initiating treatment with finerenone. Patients were generally followed up at 1, 3, and 6 months after treatment based on clinical need.

Additionally, the UACR stage and the condition of proteinuria were recorded. Albuminuria stages were defined as A1 (UACR < 30 mg/g), A2 (30 mg/g ≤ UACR < 300 mg/g), and A3 (UACR ≥ 300 mg/g). Kidney function was classified according to CKD G-stages, with G1-G4 corresponding to eGFR ≥ 90, 60–89, 30–59, and 15–29 mL/min/1.73m^2^), respectively [19]. Furthermore, disease staging was conducted based on the Mogensen criteria [21, 22]. Proteinuria was categorized as severe urine protein (urine protein semiquantitative 2 +, 3 +) or light urine protein (urine protein semiquantitative 1 + or weak positive) [19].

### Outcome measures

The primary effectiveness outcome was the change in UACR from baseline. Secondary effectiveness outcomes included changes in urinary microalbumin (mALB), urinary protein semi-quantitation, and urinary N-acetyl-β-D-glucosaminidase (NAG). Safety outcomes included changes from baseline in eGFR, serum creatinine (Scr), blood urea nitrogen (BUN), and serum potassium levels. Adverse events were recorded, including hyperkalemia (serum potassium levels ≥ 5.5 mmol/L) and a decrease in eGFR of more than 30% within 1 month.

### Statistical analysis

Quantitative variables were described using mean ± standard deviation (SD) if the data were normally distributed. Otherwise, the data were expressed as the median with interquartile range. Qualitative variables were described as frequencies (numbers and percentages). For quantitative variables, indicators at different treatment time points were compared using generalized estimating equations. Comparisons between different groups were conducted using an independent t-test or the Mann-Whitney U test. Qualitative variables were compared between groups using the chi-square test or Fisher’s exact test. For the primary effectiveness outcome, UACR, a sensitivity analysis was performed using last observation carried forward imputation (LOCF); for all other outcomes, patients with missing measurements were excluded from the corresponding analyses. Subgroup analyses were conducted based on baseline albuminuria (UACR < 300 mg/g vs. ≥ 300 mg/g) and renal function, stratified using two eGFR thresholds: (≥ 90 vs. < 90 mL/min/1.73 m²) and (≥ 60 vs. < 60 mL/min/1.73 m²). Statistical analysis was conducted using R version 3.5.1 software. The significance level for statistical tests was set at two-sided α = 0.05, with *p* < 0.05 considered statistically significant.

## Results

### Baseline characteristics and treatment pattern

A total of 127 DKD patients were included in the analysis. Patient demographics and clinical characteristics at baseline are presented in Table 1. There were 87 male patients (68.5%) and 40 female patients (31.5%), with a mean age of 57.57 ± 12.15 years and a mean disease duration of 9.05 ± 6.55 years. There were 100 cases of stage III DKD (78.7%) and 27 cases of stage IV (21.3%). According to GA staging criteria, 54 patients (42.5%) were G1A2 and 27 patients (21.3%) were G2A2. Seventy-six (59.8%) patients had hypertension and 56 (44.1%) patients had hyperlipidemia.

**Table 1:** Clinical characteristics

	All (*N* = 127)
Age, Mean ± SD (range), years	57.57 ± 12.15 (27–90)
Gender, *n* (%)	
Male	87 (68.5)
Female	40 (31.5)
Disease duration, Mean ± SD (range), years	9.05 ± 6.55 (0.2–30)
Mogensen stage, *n* (%)	
Diabetic nephropathy stage III	100 (78.7)
Diabetic nephropathy stage IV	27 (21.3)
GA stage, *n* (%)	
G1A2	54 (42.5)
G1A3	9 (7.1)
G2A2	27 (21.3)
G2A3	10 (7.9)
G3A2	7 (5.5)
G3A3	4 (3.1)
G4A2	6 (4.7)
G4A3	5 (3.9)
Missing	5 (3.9)
UACR, Median (Q1, Q3), mg/g	100 (50, 253)
30–300 mg/g, *n* (%)	98 (77.2)
> 300 mg/g, *n* (%)	29 (22.8)
mALB, Median (Q1, Q3), mg/L	131 (61, 263)
Semi-quantitative urine protein in urine routine test, *n* (%)	
3+	4 (3.1)
2+	26 (20.5)
1+	34 (26.8)
Weak Positive	21 (16.5)
Negative	39 (30.7)
Missing	3 (2.4)
NAG, Mean ± SD, U/L^*^	19.21 ± 16.68
eGFR, Mean ± SD, mL/min/1.73m^2^	85.99 ± 24.99
≥ 90 mL/min/1.73m^2^, n (%)	63 (49.6)
< 90 mL/min/1.73m^2^, n (%)	59 (46.5)
Missing	5 (3.9)
Scr, Mean ± SD, umol/L	86.90 ± 32.11
Serum potassium, Mean ± SD, mmol/L	4.17 ± 0.39
Complications and comorbidities, *n* (%)	
Hypertension	76 (59.8)
Hyperlipidemia	56 (44.1)
Diabetic peripheral neuropathy	45 (35.4)
Atherosclerosis	38 (29.2)
Hyperuricemia	34 (26.8)
Diabetic retinopathy	31 (24.4)
Finerenone dose, *n* (%)	
10 mg/day	121 (95.3)
20 mg/day	6 (4.7)
Concomitant medication, *n* (%)	
SGLT-2i	101 (79.5)
RASi	59 (46.5)
GLP-1RA	42 (33.1)
Statin lipid-lowering agents	83 (65.4)

UACR, urine albumin creatinine ratio; mALB, microalbumin; eGFR, estimated glomerular filtration rate; Scr, serum creatinine; SGLT-2i, sodium-glucose co-transporter type-2 inhibitors; RASi, renin-angiotensin system inhibitors; GLP-1RA, glucagon-like peptide-1 receptor agonist

^*^Data for urinary NAG was missing for 68.5% of patients

The median baseline UACR levels was 100 (49, 253) mg/g, 98 (77.2%) patients had a UACR of 30–300 mg/g, and 29 (22.8%) patients had a UACR > 300 mg/g. The median baseline mALB level was 131 (61, 263) mg/L. Routine urine tests revealed semi-quantitative protein abnormalities in 85 patients (66.9%), with results of 3+, 2+, 1+, or weak positive. Additionally, 39 patients (30.7%) tested negative for urine protein. The baseline mean eGFR was 85.99 ± 24.99 mL/min/1.73m^2^, with 63 patients (49.6%) having an eGFR ≥ 90 mL/min/1.73 m^2^ and 59 patients (46.5%) having an eGFR < 90 mL/min/1.73m^2^.

At the time of initiating treatment with finerenone, 79.5% of patients were receiving concomitant SGLT-2i, 46.5% were treated with concomitant RASi, and 33.1% were on a glucagon-like peptide-1 receptor agonist (GLP-1RA). Initially, a total of 121 patients (95.3%) were treated with 10 mg/day of finerenone, while 6 patients (4.7%) were treated initially with 20 mg/day. Among 121 patients who began treatment with 10 mg of finerenone daily, 26 patients increased their daily dose during treatment. Among these, one patient increased to 15 mg daily, and 25 patients increased to 20 mg daily and the mean duration of finerenone use was 86.15 ± 61.20 days after the dosage adjustment.

### Primary effectiveness outcomes

**Fig. 1:**
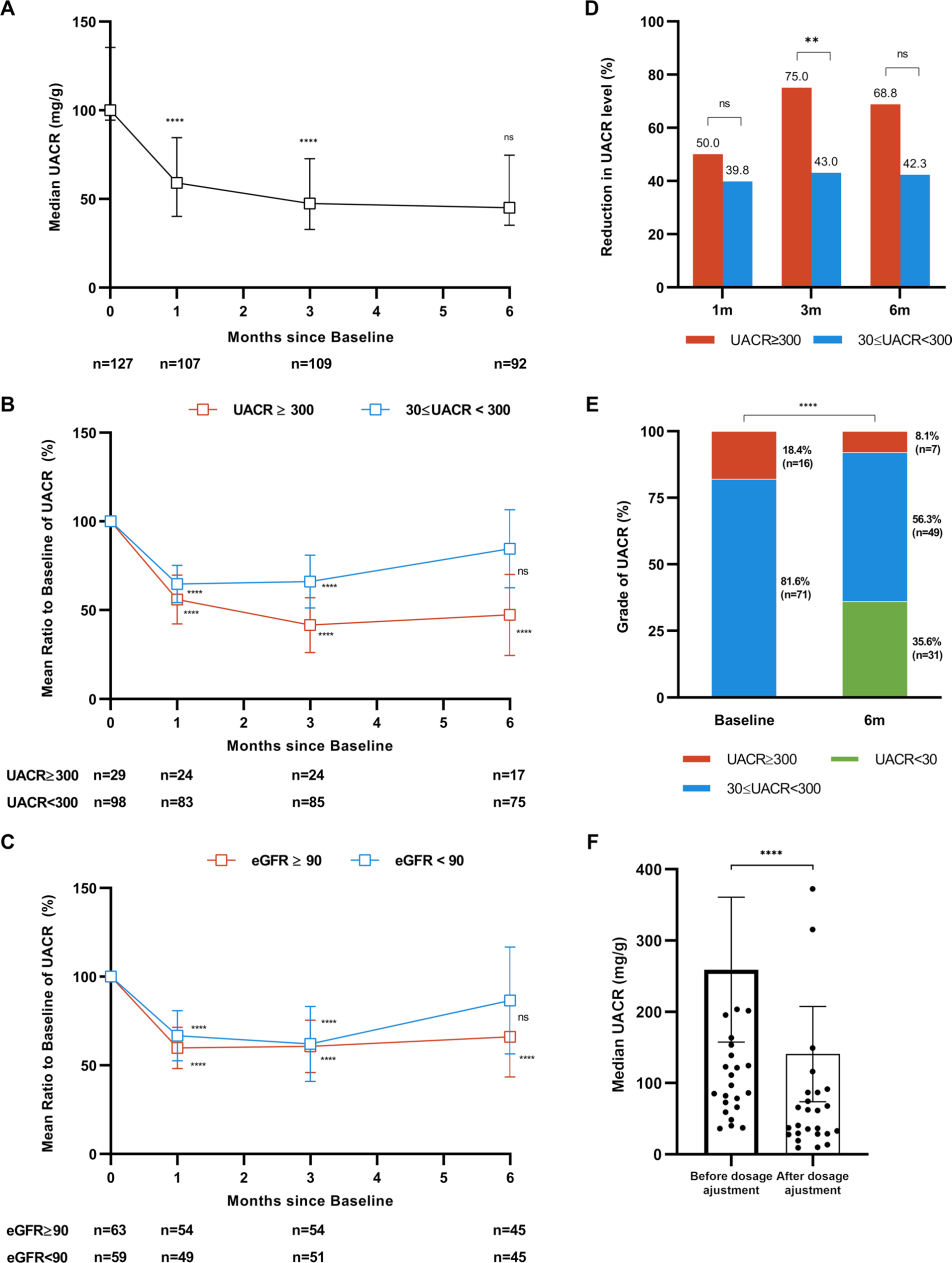
Changes in urinary albumin-to-creatinine ratio (UACR) over 6 months of finerenone treatment. (**A**) Median UACR values at baseline and at 1, 3, and 6 months (mg/g). (**B**) Mean ratio change in UACR from baseline, stratified by baseline albuminuria (UACR < 300 mg/g vs. ≥300 mg/g). (**C**) Mean ratio change in UACR from baseline, stratified by baseline renal function (eGFR ≥ 90 vs. <90 mL/min/1.73 m²). (**D**) Longitudinal shifts in UACR A-staging (A1/A2/A3) at 1, 3, and 6 months. (**E**) Composition of UACR A-staging among patients with complete 6-month follow-up. (**F**) Comparison of UACR before and after finerenone dose adjustment

UACR data were available for 127 patients at baseline, 107 at 1 month, 109 at 3 months, and 92 at 6 months. Finerenone produced a sustained reduction in albuminuria throughout follow-up (Fig. 1A), with median UACR decreasing from 100 (50, 253) mg/g at baseline to 59 (24, 122), 48 (21, 114), and 45 (22, 126) mg/g at 1, 3, and 6 months, respectively (*p* < 0.001 for 1 and 3 months; *p* = 0.097 for 6 month). Sensitivity analyses using LOCF imputation demonstrated significant reductions at all timepoints (all *p* < 0.001). Stratified analysis showed substantial UACR reductions in both A2 (*n* = 98) and A3 (*n* = 29) patients after 6 months of finerenone treatment. The mean ratio decrease in UACR at 1, 3, and 6 months was 35%, 34%, and 15% in A2 patients (*p* < 0.001 for 1 and 3 months), respectively, while it was 44%, 58%, and 53% in A3 patients over time courses (Fig. 1B, all *p* < 0.001). UACR reductions were consistent across renal function strata: patients with eGFR ≥ 90 mL/min/1.73 m² (*n* = 63) demonstrated mean ratio changes of 40%, 39%, and 34% (all *p* < 0.05), with 33%, 38%, and 13% in those with eGFR < 90 mL/min/1.73 m² (*n* = 59) (Fig. 1C, *p* < 0.001 for 1 and 3 months). Analyses using an eGFR threshold of 60 mL/min/1.73 m² showed similar patterns of UACR reduction across subgroups (Supplementary Fig. 1A).

After 1, 3, and 6 months of treatment with finerenone, UACR stage changed from A3 to A2/A1 in 12 (50.0%), 18 (75.0%), and 11 (68.8%) patients, respectively, and from A2 to A1 in 33 (39.8%), 37 (43.0%), and 30 (42.3%) patients (Fig. 1D). A total of 92 patients had complete baseline and 6-month follow-up data. Among them, 16 patients were at UACR stage A3 and 71 patients were at UACR stage A2 at baseline. After 6 months of treatment with finerenone, the proportion of patients with UACR stage A3 and that with stage A2 both decreased significantly (*p* < 0.0001), with 31 patients (35.6%) achieving a reduction in UACR to stage A1 (Fig. 1E). Among 26 patients who increased their daily dose of finerenone during treatment, patients experienced a significant reduction in UACR (Fig. 1F, *p* < 0.0001).

### Secondary effectiveness outcomes

**Fig. 2:**
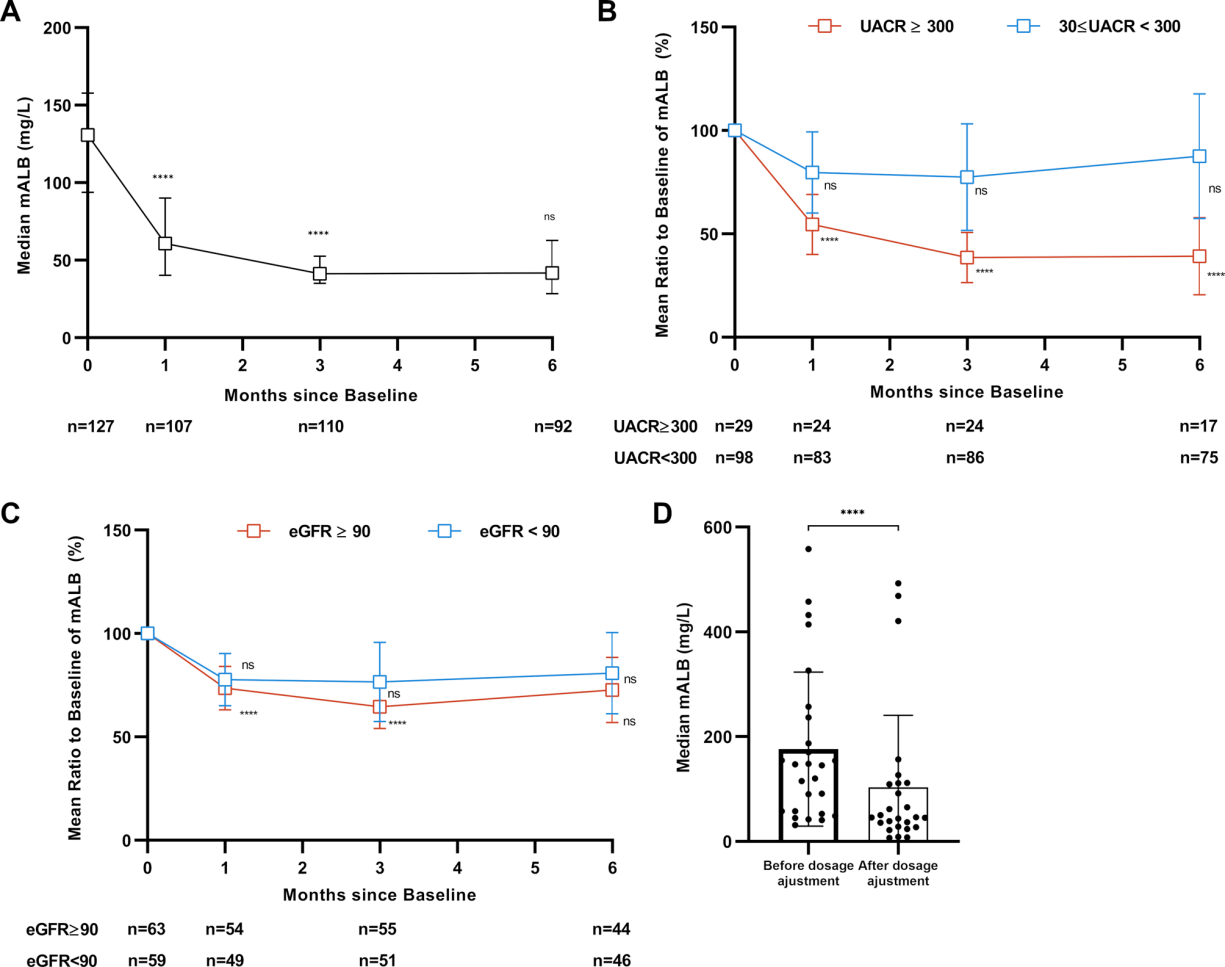
Changes in urinary microalbumin (mALB) over 6 months of finerenone treatment. (**A**) Median mALB levels at baseline and at 1, 3, and 6 months (mg/L). (**B**) Mean ratio change in mALB from baseline, stratified by baseline albuminuria (UACR < 300 mg/g vs. ≥300 mg/g). (**C**) Mean ratio change in mALB from baseline, stratified by baseline renal function (eGFR ≥ 90 vs. <90 mL/min/1.73 m²). (**D**) Median mALB values before and after finerenone dose escalation

mALB data were available for 127 patients at baseline, 107 at 1 month, 110 at 3 months, and 92 at 6 months. As shown in Fig. 2A, finerenone led to sustained reductions in mALB, with median levels decreasing from 131 (61, 263) mg/L at baseline to 61 (23, 155), 41 (22, 131), and 40 (20, 120) mg/L at 1, 3, and 6 months, respectively (*p* < 0.001 for 1 month; *p* = 0.003 for 3 month; *p* = 0.087 for 6 month). Stratified analysis showed that the mean decrease in mALB was 20%, 23%, and 12% at 1, 3, and 6 months in stage A2 patients (all *p* > 0.05), respectively, while it was 45%, 61%, and 61% in stage A3 patients at the same time points (Fig. 2B, all *p* < 0.001). mALB reductions were also consistent across renal function strata. Patients with eGFR ≥ 90 mL/min/1.73 m² exhibited mean mALB ratio changes of 26%, 35%, and 27% (*p* < 0.05 for 1 and 3 months), while those with eGFR < 90 showed corresponding ratios of 22%, 23%, and 19% at 1, 3, and 6 months (Fig. 2C, all *p* > 0.05). Analyses using an eGFR threshold of 60 mL/min/1.73 m² demonstrated similar patterns of mALB reduction across subgroups (Supplementary Fig. 1B). Among 26 patients with dosage adjustment, patients experienced a significant reduction in mALB levels (Fig. 2D, *p* < 0.0001).

**Fig. 3:**
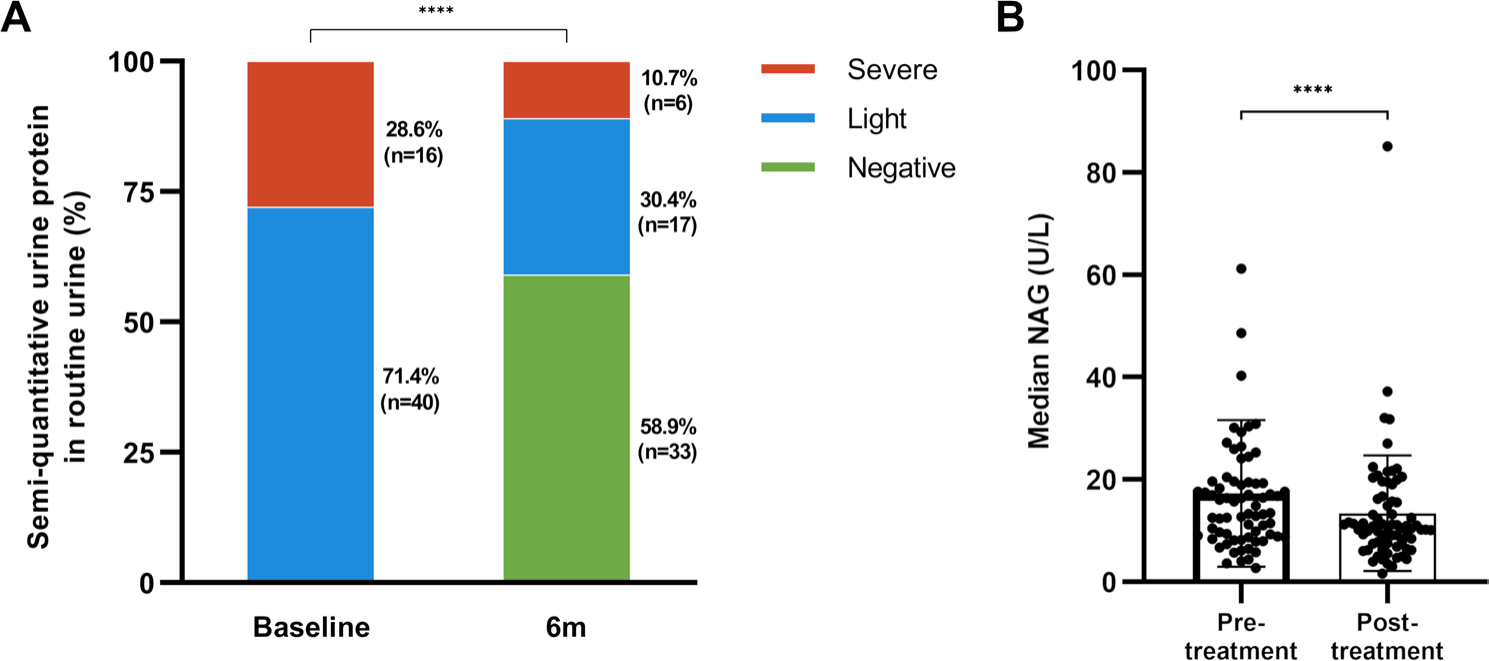
Changes in qualitative urine protein and urinary N-acetyl-β-D-glucosaminidase (NAG) during finerenone treatment. (**A**) Distribution of qualitative urine protein categories (negative, trace/1+, 2+/3+) at baseline and after 6 months of treatment. (**B**) Median urinary NAG levels at baseline and after 6 months (U/L); error bars represent interquartile ranges

Qualitative urine protein data were available for 56 patients who had positive dipstick results at baseline and completed 6-month follow-up. At baseline, 16 patients (28.57%) had severe proteinuria (2 + or 3+), and 40 (71.43%) had mild proteinuria (trace or 1+). After 6 months of finerenone treatment, the number of patients with severe and mild proteinuria decreased substantially (*p* < 0.0001), and 33 patients (58.9%) converted to a negative dipstick result (Fig. 3A). Urinary NAG was measured in 70 patients, and Fig. 3B shows a significant decline in NAG levels over the treatment period. Compared with baseline, urinary NAG concentration decreased markedly following finerenone initiation (*p* = 0.001).

### Safety analysis and adverse events

**Fig. 4:**
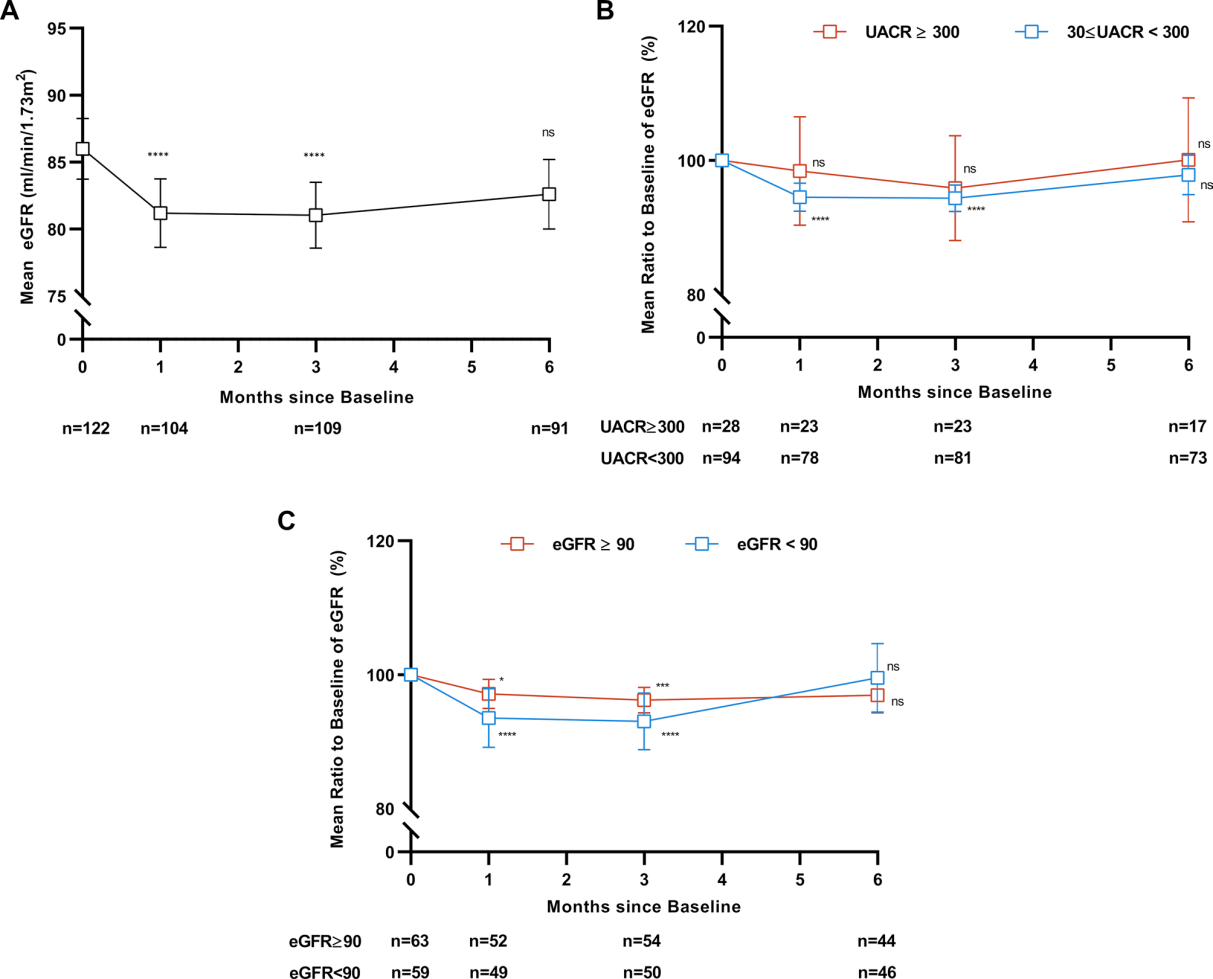
Changes in estimated glomerular filtration rate (eGFR) during 6 months of finerenone treatment. (**A**) Mean change in eGFR (mL/min/1.73 m²) from baseline at 1, 3, and 6 months. (**B**) Mean ratio change in eGFR from baseline, stratified by baseline albuminuria (UACR < 300 mg/g vs. ≥300 mg/g). (**C**) Mean ratio change in eGFR from baseline, stratified by baseline renal function (eGFR ≥ 90 vs. <90 mL/min/1.73 m²)

eGFR data were available for 122 patients at baseline, 104 at 1 month, 109 at 3 months, and 91 at 6 months. After 1, 3, and 6 months of treatment with finerenone, the mean eGFR changes from baseline of (85.99 ± 24.99 mL/min/1.73m^2^) were − 3.97 ± 9.19 mL/min/1.73m^2^ (-4.7%, *p* < 0.0001), -4.49 ± 7.92 mL/min/1.73m^2^ (-5.3%, *p* < 0.0001), and − 2.16 ± 9.75 mL/min/1.73m^2^ (-2.5%, *p* = 0.117) respectively (Fig. 4A). Stratified analysis showed that the mean decrease in eGFR was − 2.87 ± 12.41, -4.05 ± 10.11, and − 1.64 ± 10.54 mL/min/1.73m^2^ at 1, 3, and 6 months in stage A2 patients (all *p* < 0.01), respectively, while it was − 4.29 ± 8.08, -4.62 ± 7.26, and − 2.29 ± 9.63 in stage A3 patients at the same time points (Fig. 4B, all *p* > 0.05). eGFR reductions were also consistent across renal function strata. Patients with eGFR ≥ 90 mL/min/1.73m^2^ (*n* = 63) exhibited mean changes in eGFR from baseline were − 2.93 ± 7.92 (-2.8%), -3.88 ± 7.13 (-3.7%), and − 2.93 ± 8.60 mL/min/1.73m^2^ (-2.7%) at 1, 3, and 6 months after finerenone treatment, respectively (all *p* < 0.05). In the Patients with eGFR < 90 mL/min/1.73m^2^ (*n* = 59), the mean changes in eGFR from baseline were − 5.08 ± 10.34 mL/min/1.73m^2^ (-8.0%), -5.16 ± 8.72 mL/min/1.73m^2^ (-8.1%), and − 1.24 ± 10.93 mL/min/1.73m^2^ (-2.0%) at 1, 3, and 6 months of finerenone treatment, respectively (Fig. 4C, all *p* < 0.05). Over the course of 1, 3, and 6 months of finerenone treatment, eGFR levels in both groups initially declined and then raised, though there was no significant difference between the groups (*p* > 0.05). Analyses using an eGFR threshold of 60 mL/min/1.73 m² demonstrated the same pattern (Supplementary Fig. 1C).

Based on available data for Scr, with 122, 104, 109, and 91 patients examined at baseline, 1, 3, and 6 months respectively, values increased before declining. Compared to a baseline level of Scr of 86.90 ± 32.11 µmol/L, the values were 92.82 ± 33.29 µmol/L (*p* < 0.0001) at 1 month and 94.30 ± 33.75 µmol/L (*p* < 0.0001) at 3 months and then decreased to 91.44 ± 31.58 µmol/L (*p* > 0.05) at 6 months (Supplemental Fig. 2A). Similarly, for BUN, with concentrations in 121, 103, 109, and 91 patients at the same corresponding times, concentrations also trended alike. Relative to baseline BUN concentration at 6.59 ± 2.33 mmol/L, concentrations after 1, 3, and 6 months were 6.81 ± 2.35 mmol/L (*p* > 0.05), 7.25 ± 2.74 mmol/L (*p* < 0.01), and 7.16 ± 2.28 mmol/L (*p* < 0.01), respectively (Supplemental Fig. 2B). Blood pressure changes were modest. Both systolic and diastolic pressures declined slightly over follow-up (Supplementary Fig. 2C).

During the 6-month follow-up, serum potassium increased modestly from 4.17 ± 0.39 mmol/L at baseline to 4.25 ± 0.40 mmol/L at 1 month (*p* < 0.05), 4.28 ± 0.37 mmol/L at 3 months (*p* < 0.01), and 4.33 ± 0.44 mmol/L at 6 months (*p* < 0.001). The overall adverse event rate was 3.15% (4/127) during the 6-month follow-up period. Three patients experienced temporary treatment interruption due to hyperkalemia and one patient discontinued therapy because of an eGFR decline > 30% within the first month. One case of hyperkalemia occurred in stage G2A3, one in G3A2, and one in G3A3, while the single episode of eGFR decline > 30% occurred in a stage G2A3 patient.

## Discussion

This real-world study demonstrated that finerenone significantly reduces UACR, mALB and NAG levels in Chinese patients with DKD and largely preserved renal function. The anti-proteinuric effect was consistent over 6 months and more pronounced in patients with UACR stage A3 versus A2 at baseline. Importantly, these results extend the findings of major clinical trials to a population with better preserved eGFR. Furthermore, this study found a favorable safety profile in finerenone.

Inhibiting MR overactivation is considered one of the key strategies in the management of DKD [23]. Clinical studies have shown that although traditional steroidal MRAs can reduce proteinuria in patients with DKD, the risk of hyperkalemia limits their widespread clinical use [24]. Finerenone, a novel, highly selective non-steroidal MRA, distributes evenly between the heart and kidney, directly targeting cardiorenal MR overactivation. It has predominant anti-fibrotic and anti-inflammatory effects, which are crucial for renal and cardiac protection [25, 26]. Given its unique mechanism of action and favorable safety profile, finerenone has been shown to effectively reduce urinary albumin levels in patients with DKD, ensuring better outcomes for patients with this complex condition [27–29]. It has emerged as a standard of care for DKD.

The pivotal FIDELIO-DKD study [12] showed a 31% reduction in UACR after 4 months of finerenone treatment in CKD patients with type 2 diabetes, which remained stable. Similarly, the FIGARO-DKD study [14] showed a reduction in UACR of 32% in a comparable population. A key distinction in our study is that patients included in our study had lower baseline levels of UACR compared with the FIDELIO-DKD and FIGARO-DKD study populations, with median UACR of 100 (49, 253) mg/g and 77.2% of patients in stage A2. Despite this earlier disease state, the treatment with finerenone at 1, 3, and 6 months resulted in substantial mean reductions of UACR by 37%, 39%, and 27%, respectively. This demonstrates potent effectiveness even in patients with less severe albuminuria, reinforcing the FIDELITY pooled analysis results [13]. In addition, the UACR staging of patients was also shown to be improved after treatment with finerenone in this study. Of the 87 patients with complete UACR information at baseline and 6 months, 35.63% of patients achieved UACR stage A1, which indicates the significant UACR lowering effect in DKD patients. In the complete-case analysis, the reduction in UACR at 6 months did not reach statistical significance, which may be attributed to the higher proportion of missing data at this time point (27.6%) and the consequent reduction in statistical power. This was supported by the sensitivity analysis using LOCF imputation, which demonstrated significant UACR reductions at 6 months.

Beyond a renal endpoint, albuminuria reduction marks residual cardio-renal risk. It reflects glomerulotubular injury from metabolic/inflammatory stress and associates with early vascular dysfunction and arterial remodeling, even without overt cardiovascular disease [30]. Metabolic dysregulation and insulin resistance link to early vascular impairment, suggesting anti-albuminuric therapy may confer cardiovascular benefits beyond renal protection [31]. Thus, the UACR reduction in our study may indicate not only renal improvement but also mitigated systemic vascular risk in DKD.

The ARTS-DN study demonstrated a significant decrease in UACR in patients after 90 days of treatment with finerenone compared to placebo, showing a positive relationship between UACR reduction and finerenone dosage [32]. In this study, 121 patients were initially treated with a dose of 10 mg/day of finerenone; however, only 21.49% of patients had their dose increased during the study period. This might be associated with the good effectiveness of finerenone and patient financial constraints. It was found that UACR and mALB levels further decreased after increasing the dose of finerenone, a finding consistent with the results of the ARTS-DN study. This indicated that there was a positive correlation between the dose of finerenone and UACR reduction in patients with DKD in a real-world setting. To achieve better effectiveness, clinicians should enhance patient education during clinical practice and consider uptitrating the finerenone dose when appropriate, while monitoring serum potassium and eGFR levels.

mALB is one of the earliest objective indicators of diabetes-induced glomerular microangiopathy [33]. To our knowledge, previous studies have not focused on the effect of finerenone treatment on mALB levels in DKD patients. Our results indicated that after treatment with finerenone, mALB levels significantly decreased in DKD patients, with mean reductions of 26%, 31%, and 29% at 1, 3, and 6 months, respectively. Stratified analysis revealed a more substantial mALB reduction in stage A3 patients compared to stage A2 patients treated with finerenone for 1, 3, and 6 months. This indicates that, in real-world settings, the use of finerenone in patients with microalbuminuria or macroalbuminuria is associated with a decrease in mALB, yielding positive results. In addition, the study found that 58.9% of patients had negative semi-quantitative urine protein results after 6 months of treatment with finerenone, further confirming its effectiveness.

NAG is an acid hydrolase present in renal tubular lysosomes. Under normal circumstances, NAG cannot freely pass through the glomerular filtration membrane. However, glucose and lipid metabolism disorders in diabetic patients can lead to injury to renal proximal tubular epithelial cells, causing NAG to spill into the urine and resulting in increased urinary NAG levels. Therefore, NAG can serve as a marker for tubulointerstitial injury [34]. In addition, previous studies have shown that increased urinary NAG was associated with the development of macroalbuminuria and may serve as a prognostic biomarker in patients with DKD [35]. In this study, urinary NAG levels were measured in 70 patients, and it was found that finerenone significantly reduced urinary NAG levels in patients. Previous research in rat models confirmed that finerenone exerts potent anti-inflammatory [26], anti-fibrotic, and podocyte-protective effects, thereby reducing proteinuria and preventing functional and structural kidney damage in rats [36]. These mechanisms make finerenone a promising therapy for DKD. The observed reduction in urinary NAG holds particular translational significance, as tubular injury may precede detectable declines in eGFR. Emerging evidence suggests that early metabolic and inflammatory stress can induce subclinical tubular damage detectable only through sensitive biomarkers, long before conventional renal indices deteriorate [38]. Framed within this perspective, our findings not only demonstrate the tubular protective effect of finerenone but also underscore the importance of early MR blockade as a strategy to preserve renal integrity and mitigate downstream vascular consequences. This aligns with the growing recognition that targeting tubulointerstitial injury at its earliest stages may offer greater long-term cardio-renal protection.

MR overactivation plays a central role in the progression of DKD by promoting renal inflammation, oxidative stress, and interstitial fibrosis [6, 7]. Excessive MR signaling in tubular epithelial cells enhances pro-inflammatory cytokine production, activates profibrotic pathways such as TGF-β/Smad signaling, and contributes to tubular mitochondrial dysfunction and epithelial injury [6, 7]. Finerenone, a nonsteroidal selective MR antagonist, has been shown to attenuate these processes, thereby reducing glomerular pressure load and mitigating downstream tubular stress [11]. The significant decline in urinary NAG observed in our cohort is consistent with this mechanism and suggests an improvement in tubular integrity beyond the antiproteinuric effect. Notably, emerging evidence indicates that subclinical tubular injury may occur even in early metabolic or hepatic dysfunction, detectable only through sensitive biomarkers long before changes in serum creatinine or eGFR appear. For example, Scilletta et al. [37] demonstrated that patients with mild hyperbilirubinemia exhibited marked elevations in multiple tubular biomarkers despite preserved conventional renal indices, highlighting the vulnerability of renal tubules to early metabolic insults and the value of biomarker-based monitoring. This concept is further supported by recent evidence demonstrating that early metabolic and inflammatory stress can induce subclinical tubular damage, reinforcing the rationale for early intervention with agents such as finerenone to preserve renal integrity and mitigate long-term cardiorenal risk [38]. Incorporating this perspective, our findings reinforce the translational relevance of early detection and intervention in DKD, and support the concept that MR blockade may confer renal protection at a stage when tubular injury is present but still reversible.

Finerenone’s mechanism fits within the broader framework of precision cardiorenal medicine, which aims to target shared inflammatory and fibrotic pathways underlying both metabolic and renal disease. By modulating upstream MR-mediated oxidative and inflammatory signaling, finerenone offers a mechanism-based approach to early DKD intervention, particularly in patients with preserved eGFR [11]. Similar principles are reflected in emerging RNA-based lipid-lowering therapies such as inclisiran, which reduces PCSK9 synthesis via siRNA and exerts downstream anti-inflammatory and oxidative stress-modulating effects [39]. These mechanistic parallels highlight the growing convergence of metabolic and renal protection strategies and further support the potential role of MR blockade as an early, pathway-driven therapy in DKD.

In terms of safety, this study showed that 2.4% of patients experienced hyperkalemia, which was lower than the 9.7% incidence reported in the phase III clinical trials of finerenone [40]. This difference might be attributed to the fact that the patients in this study were in the early stages of the disease, as well as the combination of finerenone with SGLT-2i, which might decrease the incidence of hyperkalemia. At the start of treatment, an initial decrease in eGFR was observed in some patients. This phenomenon is a recognized hemodynamic effect of drugs that modulate glomerular hemodynamics such as RASi, SGLT-2i, and MRAs. This initial, non-progressive decline in eGFR is widely considered a protective alteration reflecting reduced intraglomerular pressure, as opposed to a sign of nephrotoxicity. It is thought to contribute to long-term kidney preservation and is an accepted, expected on-target effect of the medication [41]. Stratified analysis based on eGFR levels revealed no significant differences in eGFR changes between patients with normal renal function and those with impaired renal function. Only one patient discontinued treatment due to a decrease in eGFR of more than 30%. During the treatment, Scr and BUN levels showed an initial increasing trend followed by a decrease, with no associated adverse events. During treatment with finerenone, the patients’ blood glucose, lipid profile, and blood pressure levels remained stable. Overall, the safety profile of finerenone appears favorable in a real-world setting.

However, there are some limitations to this study. First, it was a retrospective study without a control group for comparison, which may have introduced selection bias. Second, the sample size was small, which may limit the generalizability of the findings to a broader patient population. Third, because the exact timing and duration of prior therapies such as SGLT-2i and RASi were not fully available from historical records, residual confounding from concomitant renoprotective treatments cannot be excluded. Fourth, albuminuria data were not available for all patients at each follow-up time point, which may introduce informative loss to follow-up bias. However, a sensitivity analysis using LOCF imputation was performed to minimize this concern. Finally, the evaluation period was limited to 6 months, assessing only the effectiveness and safety of finerenone in patients with DKD. Future real-world studies should involve a larger sample size, a longer observation period, and a focus on the long-term outcomes of finerenone in patients with DKD to further evaluate its comprehensive effectiveness and safety in clinical practice.

## Conclusion

This real-world study demonstrated that finerenone is effective and safe in reducing proteinuria in patients with DKD. It demonstrated a greater reduction in UACR and mALB in stage A3 patients compared to stage A2 patients. However, adverse events such as hyperkalemia and acute declines in renal function require careful monitoring.

## Supplementary Information

Below is the link to the electronic supplementary material.

Supplementary Material 1: Supplementary Fig. 1 Subgroup analyses of treatment effects stratified by baseline renal function (eGFR ≥ 60 vs. <60 mL/min/1.73 m²). (A) Mean ratio change in UACR from baseline at 1, 3, and 6 months. (B) Mean ratio change in mALB from baseline. (C) Mean ratio change in eGFR relative to baseline.

Supplementary Material 2: Supplementary Fig. 2 Changes in serum creatinine (Scr), blood urea nitrogen (BUN), and blood pressure during finerenone treatment. (A) Mean change in Scr (µmol/L) from baseline at 1, 3, and 6 months. (B) Mean change in BUN (mmol/L) over the same time points. (C) Mean change in systolic and diastolic blood pressure (mmHg) during follow-up.

## Data Availability

The original contributions presented in the study are included in the article and supplementary material. Further inquiries can be directed to the corresponding author.

## Abbreviations

BUN: Blood urea nitrogen
CKD: Chronic kidney disease
DKD: Diabetic kidney disease
eGFR: Estimated glomerular filtration rate
ESRD: End-stage renal disease
GLP-1RA: Glucagon-like peptide-1 receptor agonist
UACR: Urine albumin-to-creatinine ratio
mALB: Microalbumin
MR: Mineralocorticoid receptor
MRA: Mineralocorticoid receptor antagonists
NAG: N-acetyl-β-D-glucosaminidase
RASi: Renin-angiotensin system inhibitors
RCTs: Randomized controlled studies
Scr: Serum creatinine
SD: Standard deviation
SGLT-2i: Sodium-glucose co-transporter type 2 inhibitors
T2DM: Type 2 diabetes mellitus
