# Supplementary figures and images for: Real-world effectiveness and safety of finerenone in diabetic kidney disease with preserved eGFR: a retrospective study in China
Source: BMC Nephrol. 2026 May 12;27:403. doi: 10.1186/s12882-026-05035-4 (PMC13339712; doi:10.1186/s12882-026-05035-4)

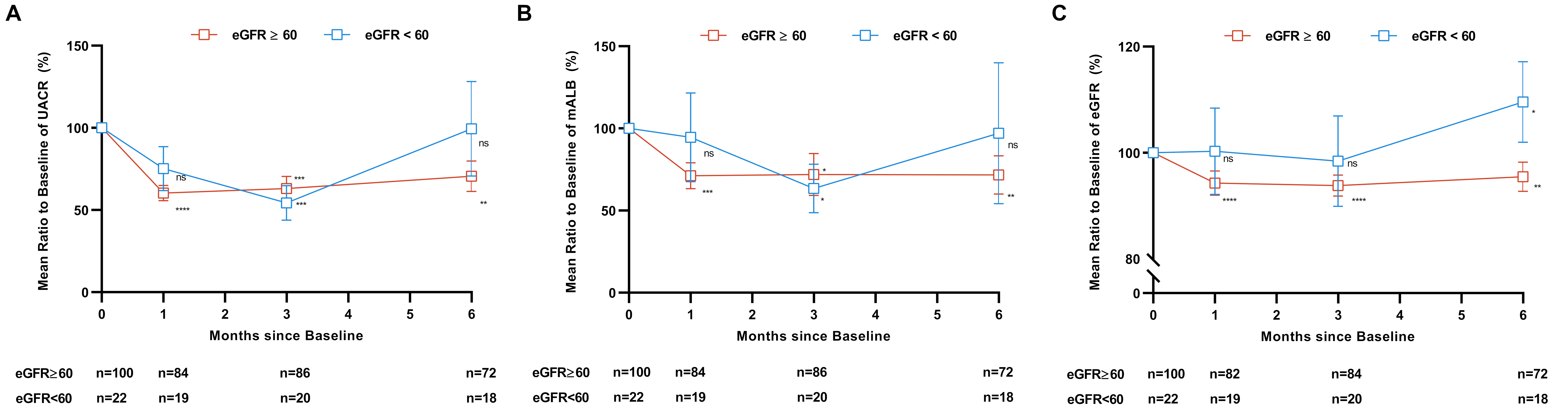

Supplement: Supplementary file 1 — Supplementary Material 1: Supplementary Fig. 1 Subgroup analyses of treatment effects stratified by baseline renal function (eGFR ≥ 60 vs. <60 mL/min/1.73 m²). (A) Mean ratio change in UACR from baseline at 1, 3, and 6 months. (B) Mean ratio change in mALB from baseline. (C) Mean ratio change in eGFR relative to baseline. [file 12882_2026_5035_MOESM1_ESM.tif]

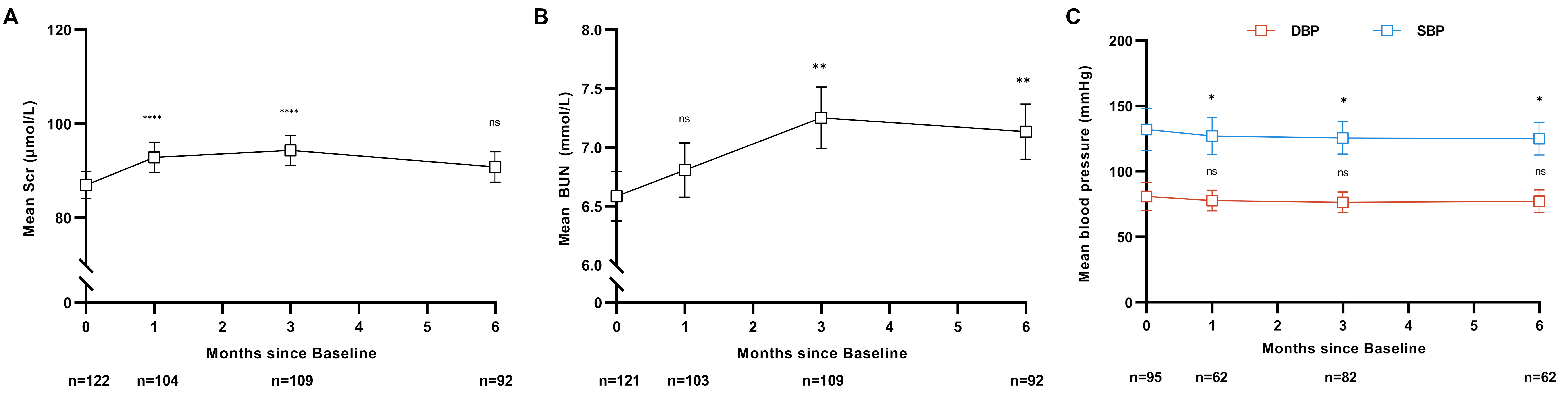

Supplement: Supplementary file 2 — Supplementary Material 2: Supplementary Fig. 2 Changes in serum creatinine (Scr), blood urea nitrogen (BUN), and blood pressure during finerenone treatment. (A) Mean change in Scr (µmol/L) from baseline at 1, 3, and 6 months. (B) Mean change in BUN (mmol/L) over the same time points. (C) Mean change in systolic and diastolic blood pressure (mmHg) during follow-up. [file 12882_2026_5035_MOESM2_ESM.tif]
